# Variety in Diet

**Published:** 1876-10

**Authors:** Alton Cheswicke


### *VARIETY IN DIET,*

### IN ITS RELATIONS TO HEALTH.

By Alton Cheswicke.

The civilized man, being no longer dependent upon the chances of the chase, is enabled, through the agency of the grazier and butcher—provided, of course, that he has the requisite amount of legal tender—to command not only meat, fresh or salt, but also *fat* meat every day in the year, if he wants it; and we have only to stroll through the streets between the hours of five and seven in the afternoon of a broiling, blistering day in July or August, and note the number of butcher shops that are open and the customers that throng them, to obtain a realizing sense of the amount of meat that is consumed in our cities during midsummer, and for the evening meal at that! The majority of city people evidence the possession of a small remnant of common sense, by bestowing their patronage almost wholly upon *fresh* meat, usually confining themselves to beef and mutton; although here and there a misguided individual may be found who includes fresh and salted pork, ham, and other smoked and salted meats in his midsummer bill of fare, and takes the consequences. But in the country—always inseparably connected in the minds of dwellers in town with the idea of juicy fruits, crisp, dewy vegetables, and everything that is fresh and refreshing during the fiery summer season,—in the country, may be found beings so lost to all sense of the fitness of things, so ignorantly or so perversely rebellious against the laws of nature, as to feed, or rather poison, their families all through the languid spring and hot summer months as well as at other seasons, on *pork*—and not only that, but *salt* pork from last winter's brine; and not only that, but *fat* salt pork, very often fried at that! Shades of Pomona, Ceres, and all the countless and benignant influences that preside over golden harvest fields and bending orchards! arise in your righteous indignation and avenge this deadly affront put upon your several ministrations to man—this apostate desertion of your altars at the season when ye should reign supreme. But we have no need thus to cry out for retribu tion; nature, who never forgets or overlooks any infringement of her laws, will visit upon every such culprit's head a penalty worthy of the offense.

Now, we are actuated by no sentiment of personal hostility or undue prejudice against the pig as an individual. We recognize his usefulness to man in his proper sphere—within which, however, we insist he shall remain—and we can look with a tolerant eye, though we will not promise to partake—upon pork, even *fat* pork for the out-door worker, during the winter season when the cold is extreme; but in the spring, and especially in the summer time, the very name of pork is, and should be, an abomination to every rightly constituted individual, and our acquaintance with the worthy creature that supplies it, must exist, if at all, upon purely sociable and never upon comestible grounds.

Let us, then, who possess advantages for so doing so much superior to those of poor Lo., follow his excellent example, and banish meat from our table altogether during the summer season, when there is so much to be had and enjoyed that is vastly better.

Right here, perhaps, some one will start up and say, "Why, that is just what we do, and hundreds of other well regulated families who, like us, confine themselves to a fish diet in the spring, and who seldom or never put meat upon their tables the whole summer through, and who are yet but little, if at all, the better in health for so doing.

Granted that they are only a little better—for that they are to some extent bettered thereby, we insist—it will be easy to demonstrate the wherefor without in the least invalidating our position. There are a great many people who—figuratively speaking, at least, take out of one pocket only to put with a more liberal hand, if possible, into the other; and it will not be hard to prove that the majority of those who complain that they derive but little benefit through abstaining from a meat diet during the summer months, nullify the good effects of their abstemiousness in this respect by undue indulgence in some equivalent for meat that is just as bad.

How many American families are there, for instance, who do not use butter—which is another great American staple—once or twice, if not three times a day *the year round*, without much, if any, regard to the nature of the items of food that may accompany it at each meal? Now, butter being the essence of milk, which is in itself the essence of meat, is only meat itself in another and more concentrated form, and consequently possesses to a great degree all the qualities which render meat nutritious or injurious. In a word, butter is unquestionably a kind of grease; and it has been demonstrated that there are times and seasons during the year when the human system has no need of grease in any form—quite the contrary. And to impose upon the system any elements which it does not require, is to inflict upon it an injury.

But if those who thus use butter in lieu of meat or any other form of fat, are convicted of folly, and, hygienically speaking, misdemeanor, what shall be said of those who, adding enormity to enormity, heap grease upon grease? Some article of food, for instance, having been prepared by being fried up or warmed over in fat of some kind—suet, lard, gravy or butter—comes upon the table saturated, of course, with grease. Now, common sense and common prudence would admit that here is certainly grease enough for one meal's consumption, if not too much, indeed; and would suggest, moreover, the advisability, if not the necessity, of an absorbant of some kind to help dispose of it all, or counteract what might otherwise be the bad effects of so much of one article. Such an absorbant, fortunately, is to be found upon every civilized table in the form of dry bread; but instead of being eaten dry, as if there were not fat enough already to be disposed of, it is in too many cases liberally spread with butter—another form of grease—and thus its usefulness as a counteractant is wholly destroyed, and it is but made the vehicle for increasing what is already much too abundant. Who among the readers of Hall's Journal has not seen this outrage on good taste—to stigmatize it no further—perpetrated again and again at the tables of those who imagine they know how to live; butter being eaten with gravy, fat meat, and all manner of dishes prepared by the aid of suet or lard! In ancient times metal upon metal was considered false heraldry, and surely, in these later times, grease upon grease is still worse hygiene. It is unpermissable in winter—how much more, then, in spring and summer!

We must protest again that we have no intention or wish to banish butter altogether from any one's table; for although not interested in butter as an article of merchandise, we hold it to be a valuable as it is a savory food. We indorse a moderate and judicious use of it during the fall and winter; we believe that it advantageously takes the place of other and grosser forms of fat less suited to the requirements of the *civilized* man, and that it was meant to do so. We claim that the system has need of, and therefore demands it at certain times. We would not totally prohibit its use even during spring and summer; but we leave it to the good sense of any and every one who is convinced of the imperative need of the system for variety and change, to decide whether the constant use of that one article, butter, even though in but small quantities, at every meal in the day, and every day in the week, month in and month out, year after year, is likely to be productive in the end of more beneficial than injurious results.

To be Continued.

